# Urinary miRNAs as a Diagnostic Tool for Bladder Cancer: A Systematic Review

**DOI:** 10.3390/biomedicines10112766

**Published:** 2022-10-31

**Authors:** Anna Maria Grimaldi, Cristina Lapucci, Marco Salvatore, Mariarosaria Incoronato, Maurizio Ferrari

**Affiliations:** 1 IRCCS SYNLAB SDN, Via Gianturco 113, 80143 Naples, Italy; 2 Genetic Unit, Synlab Italia, 25014 Castenedolo, Italy

**Keywords:** miRNAs, bladder cancer, biomarker, diagnosis, urine, supernatant, pellet, sediment, extracellular vesicle

## Abstract

Bladder cancer is the 10th most common cancer type worldwide. Cystoscopy represents the gold standard for bladder cancer diagnosis, but this procedure is invasive and painful, hence the need to identify new biomarkers through noninvasive procedures. microRNAs (miRNAs) are considered to be promising diagnostic molecules, because they are very stable in biological fluids (including urine) and easily detectable. This systematic review analyses the power of urine miRNAs as bladder cancer diagnostic markers. We conducted this systematic review according to the Preferred Reporting Items for Systematic Reviews and Meta-Analyses (PRISMA) statement. A total of 293 records related to miRNAs and their diagnostic significance in BC were retrieved from the PubMed and Embase databases. A systematic search of the literature was performed, and a total of 25 articles (N = 4054 participants) were identified and reviewed. Although many of the selected studies were of high scientific quality, the results proved to be quite heterogeneous, because we did not identify a univocal consensus for a specific miRNA signature but only isolated the signatures. We did not identify a univocal consensus for a specific diagnostic miRNA signature but only isolated the signatures, some of them with better diagnostic power compared to the others.

## 1. Introduction

According to Globocan 2020 [1], bladder cancer (BC) is the most common malignancy of the urinary tract and the 10th most common cancer worldwide, with 570,000 incident cases and 200,000 deaths per year. It represents the 6th most malignant cancer in men, with 440,000 incident cases and 158,000 deaths per year, and 17th in women, with 130,000 incident cases and 50,000 death per year [1]. BC ranks third in Egypt and Tunisia, fourth in Libya and Jordan, and fifth in Europe and Canada. Almost 80% of bladder cancers are diagnosed as non-muscle invasive bladder cancer (NMIBC) [2], with a recurrence rate of 60% within 1 year of the first diagnosis [3]. The diagnosis of BC is incidental and subsequently discovered at clinical manifestation as hematuria, flank pain, or a palpable mass [4]. A diagnosis can be made with an ultrasound scan (US) and cytology evaluation, but the gold standard for a confirmative diagnosis is a cystoscopic examination. Even if the US and cytology evaluation are easy procedures, not invasive and at a reduced cost, the sensibility and sensitivity are not so high with the limited reproducibility [5]. The cystoscopic examination is an invasive procedure; limited by variables related to low cellularity, infections, and artifacts caused by the maneuvers [6]; and should be repeated every 3–6 months for five years, according to the stage, grade, and recurrence [7]. Therefore, there is a high interest in finding new biomarkers to increase the sensibility and specificity using less invasive methods. Thanks to the increasing knowledge of liquid biopsy in oncology, many works have focused on the discovery of biomarkers in biological fluids (such as blood, plasma, urine, liquor, and saliva) not only to monitor cancer evolution but also for diagnostic purposes, avoiding the limitations of invasive procedures [8,9].

In this framework, microRNAs (miRNAs), a large family of small (20–22 nucleotides) noncoding RNAs, may be helpful as diagnostic and prognostic biomarkers in different human pathologies [10,11,12]. They regulate gene expressions at the post-transcriptional level, as a single miRNA can target and regulate up to hundreds of genes [13], each of which could be involved in biological pathways pathogenically disrupted in a diseased patient [14]. Although the alteration of miRNAs has not been fully elucidated in the bladder, miRNAs are deregulated in bladder cancer and have been seen to promote cell proliferation and progression through epithelial-to-mesenchymal transition and inhibit apoptosis [15,16]. Therefore, thanks to their high stability in biological fluids, miRNAs represent one of the most promising biomarkers for cancer detection, including bladder cancer [17]. Here, we provide a systematic overview to collect and summarize the current state-of-the-art trials on urine miRNAs as promising biomarkers for a BC diagnosis.

## 2. Materials and Methods

### 2.1. Search Strategy and Eligibility Criteria

**Figure 1:**
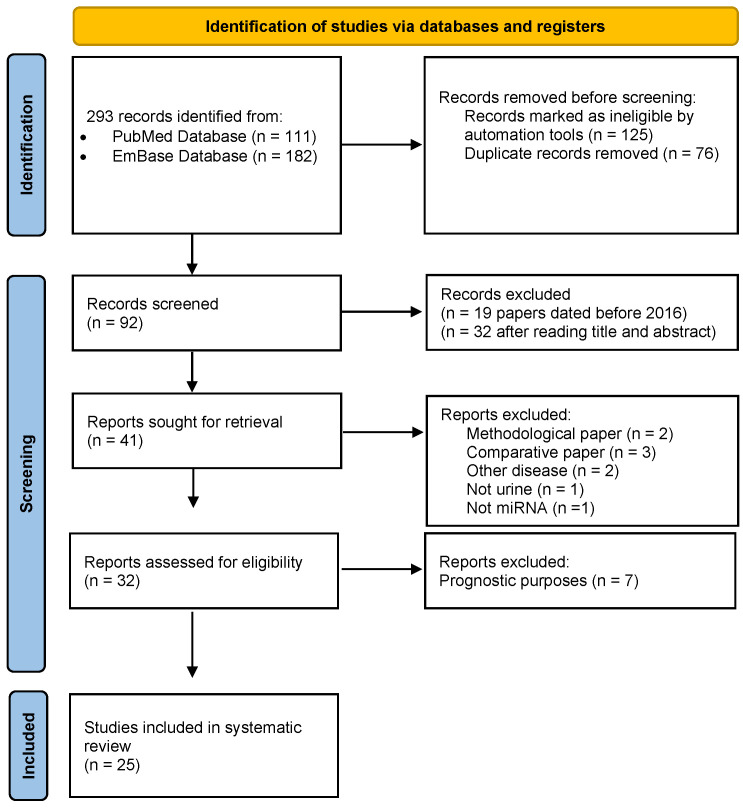
PRISMA 2020 flow diagram, which included searches of the databases.

A systematic literature search was conducted to identify studies reporting the urinary expression of one or more miRNAs as a noninvasive tool for the diagnosis of BC. We conducted this systematic review following the Preferred Reporting Items for Systematic Reviews and Meta-Analyses (PRISMA) statement (for more details, see the PRISMA Checklist in Supplementary Materials: Listing S1, Table S1). As shown in Figure 1, a comprehensive search was carried out in two scientific electronic databases (PubMed and EMBASE) for articles published up to and including July 2022. The search results were tabulated, and duplicates were removed. Three authors independently reviewed the articles for eligibility from the titles and abstracts. Then, the full text of each article was retrieved and assessed for final eligibility independently by any three authors. Any discrepancies between the authors concerning eligibility were resolved by consensus among all the authors. The articles that met the following inclusion criteria were considered: (1) sample size of 10 patients and above, (2) urine collection, (3) report of the expression levels of miR, (4) report of the diagnostic performance of proposed biomarkers, (5) available in the English language, as well as (6) randomized controlled or cohort studies, and (7) preprints or published papers. On the other hand, we excluded non-English papers, reviews, metadata, single case reports, letters to the editor, methodological studies, and papers exclusively focused on the mechanistic involvement of miRNA in BC (in vitro studies). Although our queries found studies published since 2010, a meta-analysis with a similar topic was published in 2016 [18], so we decided to exclude all papers dated before 2016.

### 2.2. Data Extraction and Collection

After selecting all collected records, three investigators summarized data that met the inclusion criteria into a customized Excel spreadsheet. A fourth author checked the extracted data for completeness and accuracy. Any disagreements were resolved by consensus among the authors. Data extraction from the selected publications was done using a standardized table by three authors independently. For each study, the extracted data are reported in Table 1 as follows: Reference, Publication Year, Other biospecimen, Urine Specimen, Centrifuge Protocol, Study Cohort, Method, Reference Gene, miRNAs, Multiple-miRNAs Signature, and Diagnostic Power (AUC).

The quality of each article was evaluated by the revised quality assessment of diagnostic accuracy studies (QUADAS-2) checklists [19]. All disagreements about the collected data were adequately debated by the investigators, and they arrived at a final consensus.

## 3. Results

### 3.1. Study Selection

A flowchart of the literature search and the detailed selection process of the articles are reported in Figure 1. A total of 293 records related to miRNAs and their diagnostic significance in BC were retrieved from public databases. Of these, 125 papers were removed, because they were not original articles, and 76 reports were excluded as duplicates. Next, the remaining 92 records were screened for the title, abstract, and publication year. A total of 19 records were excluded, because they dated before 2016, and 32 were not eligible after reading the title and abstract. Thus, a total of 41 records were selected for full-text reading. Of these, nine studies were excluded, because they were methodological/comparative/methylation/on other diseases and not on urine biofluid. From the remaining 32 reports, an additional 7 studies were excluded, because they had prognostic purposes only. Finally, we identified 25 records for our systematic study.

### 3.2. General Findings

The main features of each selected study are shown in Table 1. The eligible 25 articles were published between 2016 and 2022. About 42% of the studies were performed in Europe, as well as in Asia, 8% in Africa, 4% in America, and 4% in Oceania, with a total population of N = 4054 enrolled subjects. Of the reviewed articles, the largest cohorts were enrolled in two Asian studies [20,21], with a total of 1074 subjects.

Approximately 48% of the studies did not report any specification about the tumor classification, whereas 52% specified if the BC tumor was non-muscle invasive bladder cancer (NMIBC) or invasive muscle bladder cancer (MIBC). About 32% of the studies screened urine plus other biological samples such as blood [22,23,24], tissue [24,25,26,27,28,29], or cell lines [25,29]. Among the 25 selected studies, supernatant and whole urine were the eligible choices (*n* = 8 and *n* = 8, respectively; see Section 3.1 and Section 3.2), whereas urine sediments and urine exosomes were used in a smaller number of studies (*n* = 4 and *n* = 5, respectively; see Section 3.3 and Section 3.4). Most of the studies validated by reverse transcription polymerase chain reaction (RT-qPCR) an a priori signature of miRNAs, and only a small portion of studies made use of high-throughput approaches such as next-generation sequencing (*n* = 5) and microarrays (*n* = 7) to find the best miRNAs for diagnostic purposes (see the Method column in Table 1. 

### 3.3. Quality of the Selected Articles

**Figure 2:**
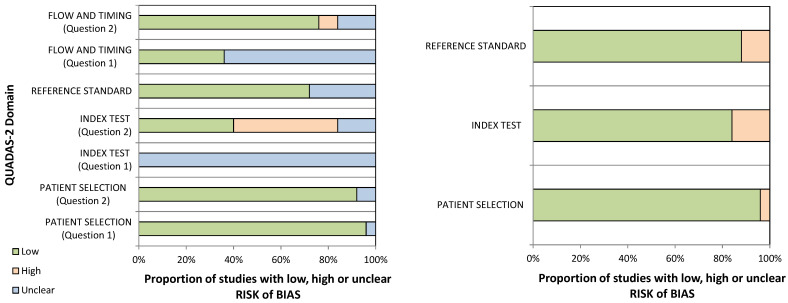
Graphical display for the QUADAS-2 results.

QUADAS-2 was used to assess the diagnostic accuracy of the 25 eligible studies. The risk of bias and applicability concerns graph for the included articles is shown in Table 2 and Figure 2. We found that all 25 reports met the criteria for a high-quality score (Figure 2). Specifically, they were well described and adequately answered the quality questions. Regarding the risk of bias, more than 90% of the studies had a representative spectrum of patients, including clear selection criteria. However, none of the selected studies clarified (unclear) if the index test results were interpreted according to the double-blind procedure: means without knowledge of the reference standard result. About 60% of the studies did not specify the threshold used for the index test, and the reference standard used to classify the target condition was unclear for 28% of the studies. Still, the appropriate time interval between the index test and the reference standard were provided only for 36% of the selected papers. Regarding the applicability concerns, all studies (100%) matched the reviewer’s questions (Table 2 and Figure 2).

### 3.4. miRNAs Identified as Bladder Cancer Diagnostic Markers

As reported in Table 1 (column miRNA), 82 different markers were associated with BC disease: about 78% were found to be upregulated, and the remaining 22% were reported to be downregulated. As reported in Table 3, these miRNAs were clustered based on the frequency of their finding in the manuscripts. The most frequently recurring BC-associated miRNA was miR-96 identified that was found upregulated and correlated with a BC diagnosis in three studies [23,25,43]. Then, 17 miRNAs, each of them, were found deregulated in more than one study (Table 3). We expected each of the 17 miRNAs to have the same trend in the two studies where they were found to be BC-associated; this was true except for miR-141-3p, miR-185-5p, and miR-200a. Specifically, they were found upregulated by [31,36,43] and downregulated by [21,36,37]; even if the isoform of miR-200a was not specified [43]. Moreover, as indicated in Table 1, we found that the diagnostic power of miRNAs in terms of AUC (Area under the ROC Curve) was determined in 14/25 studies (56%) and 7/25 studies (28%), as related to a multiple or single diagnostic miRNA signature, respectively. Instead, in 4/25 studies (16%), the identified miRNAs were not associated with any diagnostic power but only to a statistical significance expressed as a *p*-value able to discriminate BC subjects from healthy controls. In addition, it was interesting to find out that, to improve the diagnosis of BC, eight studies [20,21,23,25,27,29,37,40] combined the diagnostic power of miRNAs with other biomarkers (proteins, microscopy, cytology, and lncRNAs).

The discussion section (Section 4) details the results of the 25 studies, their similarities, and their differences.

## 4. Discussion

Liquid biopsy refers mainly to blood, plasma, urine, and saliva. Its analysis represents a noninvasive method to isolate biomarkers (circulating tumor cells, cell-free tumor DNA, cell-free tumor RNA, proteins, peptides, and metabolites) for diagnostic and prognostic information. Being the blood closely correlated with markers of diseases, it represents the main biological sample used to identify new molecules able to improve the management of oncological patients. Nevertheless, being in direct contact with BC, the urine samples could represent the ideal source for investigating new noninvasive biomarkers, including miRNAs. As for the blood [44,45,46], the use of urine for diagnostic purposes has some limitations, and knowing them must be crucial before their choice. Therefore, it is necessary to know that urine: (i) is an unstable fluid that changes composition as soon as it is eliminated through micturition [47], (ii) accumulated overnight in the bladder is more concentrated, detecting trace amounts of molecules that could be lost in more diluted samples [48], (iii) may contain the presence of bacteria, so its contamination may produce various inaccurate results, and (iv) includes sediment and a supernatant that differ from each other. As the sediment and supernatant have different molecular components, the question is: which one between them could represent the more appropriate sample to identify miRNAs for diagnostic purposes? What if whole urine were used for a miRNA analysis?

The 25 eligible studies selected in this systematic review have in common the same goal: “to identify circulating miRNAs for BC diagnosis”; nevertheless, they are somewhat heterogeneous in the choice of screened biospecimens. In fact, eight out of twenty-five studies (32%) focused on whole urine, eight were on supernatants (32%), five were on urine sediments (20%), and four on urinary exosomes (16%). Based on this, we decided to discuss the results of the 25 studies by dividing them into four subgroups: whole urine, urine supernatant, urine sediment, and urine exosome.

### 4.1. miRNA Biomarker Isolated from Whole Urine

The use of whole urine has emerged as one of the two most widely employed methodological strategies to isolate miRNAs linked to BC (8/25 studies) [16,24,31,32,35,38,39,42]. Querying Table 1, the first evident finding regards the study cohorts. Assuming that the use of two separate cohorts (discovery and validation cohorts) boosts the probability of finding effective diagnostic biomarkers, we found that, only in 3/8 of the studies, the authors took advantage of a discovery cohort and a validation cohort [16,24,42]. The total cohort for these three studies was 302 BC patients and 553 healthy controls, while, for the remaining five studies [31,32,35,38,39], it was 349 BC patients and 461 healthy controls. However, considering the mean value of the patients in two sets of studies, it can be seen that the sample size better supported the three studies’ comprehensive discovery and validation cohorts rather than the five with a unique study cohort (BC 100 and Controls 184 vs. BC 70 and Controls 92, respectively).

Jen-Tai Lin and Kuo-Wang Tsai performed a NGS analysis on 10 urine samples of BC patients and 10 healthy subjects [24]. They identified 50 circulating urinary miRNA differentially expressed in BC vs. healthy controls. These miRNAs, analyzed according to a pathway enrichment analysis, appeared to be involved in cancer-related signaling pathways (proteoglycans in cancer, MAPK, TGF-beta, FoxO, colorectal cancer, cellular senescence, the adherens junction, PI3K-Akt, Hippo, autophagy, focal adhesion, and ErbB). Among all identified miRNAs, the authors selected five upregulated (let-7b-5p, miR-146-5p, miR-149-5p, miR-423-5p, and miR-193a-5p) for further examination in BC. By an in silico analysis (The Cancer Genome Atlas database), they found that these miRNAs were higher in BC tissues compared to normal tissues and that high levels of miR-149-5p and miR-193a-5p were associated with poor overall survival. Then, this miRNA signature was validated in a separate cohort of 90 healthy controls and 70 BC patients, and, except for miR-193a-5p (*p*-value = 0.11), all miRNAs were found significantly (*p*-value ≤ 0.001) overexpressed in the urine of BC patients compared to healthy donors. Even if this study reported interesting results, the authors did not determine any AUC value, so the diagnostic power of let-7b-5p, miR-146-5p, miR-149-5p, and miR-423-5p cannot be compared with those of the miRNAs identified in the other studies.

The study of [16], by proofing 384 miRNAs (microarray) from urine samples of a discovery cohort (12 BC and 8 healthy donors), selected and validated 14 miRNAs in an independent cohort (115 BC and 87 healthy donors). ROC curves for the prediction of BC using the six miRNA signatures were able to discriminate BC from the controls well (AUC = 0.883). This signature included three down-expressed (let-7c, miR-148a, and miR-204) and three overexpressed (miR-135a, miR-135b, and miR-345) miRNAs. This panel was most accurate in diagnosing NMIBC (AUC = 0.93) but also able to identify MIBC (AUC = 0.88) and MIBC (AUC = 0.91). 

The study of [42], differently from the above, did not use high-throughput methods (NGS or microarray). The authors chose the a priori signature of 12 miRNAs, known to be involved in epithelial cancer carcinogenesis, to assess their usefulness for a BC diagnosis. These miRNAs were screened in the urine of a discovery cohort including 81 subjects. Using a machine learning approach, they identified six miRNAs (miR-16, miR-200c, miR-205, miR-21, miR-221, and miR-34a) with the best diagnostic performances (AUC = 0.85). This signature was then validated in an independent cohort (AUC = 0.74), allowing the detection of larger tumors (AUC = 0.81) and tumors with higher T-stages (AUC = 0.92). This study also raised interest in choosing osmolarity as the methodological approach to normalize miRNA expression data. Although these three studies demonstrated robust results, none of them shared miRNAs in common. Therefore, these three studies highlighted three different miRNA signatures for a total of 16 miRNAs linked to a BC diagnosis. The best diagnostic power for discriminating BC from healthy controls seems to be that of [16], with an AUC = 0.88. One of the discrepancies we found in almost all the studies analyzed in this systematic revision, which we also highlight in the other sections, is the choice of the reference gene for data normalization. It is necessary to point out that these three studies did not use the same reference genes, and it is known that test results vary according to the internal control used. In the manuscript of [24], we did not find any reference they used. In this study, [16], the authors used RNU48 and miR-103, and Sapre and colleagues [42] used osmolarity to normalize the test results. 

The remaining five studies [31,32,35,38,39] identified miRNA signatures with a diagnostic power lower than AUC = 0.8, and they did not validate the data using a separate cohort (validation cohort). Among them, the study by [38] enrolled the highest cohort, but no AUC value was indicated for the diagnostic power of miR-126 in discriminating BC patients from healthy donors. In addition, these two studies [31,32] used 5S rRNA as a reference gene, [35,38] used RNU6, and [39] used miR-21-5p. Therefore, evaluating all miRNAs isolated in these eight studies, we found that only three of them were in more than one study and showed the same trends (Supplementary Table S2): miR-205 was found upregulated both by [31] and by [42], miR-146a-5p was found upregulated by both [24] and by [39], and miR-21 was found upregulated by both [31] and by [42]. In contrast, even though miR-141 was identified in two studies, its trend was the opposite: upregulated by [31] and downregulated by [32].

### 4.2. miRNA Biomarkers Isolated from Urine Supernatant

A total of 8/25 studies [21,26,30,33,34,37,40,41] used the urine supernatant as a biospecimens for miRNA selection, and 6 of them [21,30,33,37,40,41] took advantage of both a discovery cohort and a validation cohort (Table 1). Overall, these six studies enrolled 198 BC and 133 controls for the discovery cohort, whereas 862 BC patients and 732 controls were enrolled for the validation cohort. The sum of both cohorts was impressive: 1060 BC patients and 865 controls. The most contributing to these numbers become undoubtedly from two studies [30,37]. Alone, they collected a total of 567 supernatants from the urine of BC patients and 507 supernatants from the urine of healthy controls, and in both studies, the authors used high-throughput methods (NGS or microarray) for the selection of urine supernatant miRNAs differently expressed between affected and unaffected subjects. The results of [37] and those of [30] were very interesting in terms of their AUC values. Nevertheless, even if the two groups used cutting-edge methods on a vast study cohort, their findings were discordant; they isolated two different miRNA signatures with high diagnostic powers. Du and colleagues [37], by using a multivariate logistic regression model, identified a seven-miRNA panel (miR-7-5p, miR-22-3p, miR-29a-3p, miR-126-5p, miR-200a-3p, miR-375, and miR-423-3p) that provided a very high diagnostic accuracy (AUC of 0.923 and 0.916 in the training and validation cohorts, respectively) compared to traditional urine cytology. They demonstrated that this panel also works very well for the tumor stage (Ta: AUC = 0.864, T1: AUC = 0.930, and T2–T4: AUC = 0.978). The seven miRNAs were found deregulated in a different trend. Those upregulated in BC patients were miR-7-5p, miR-22-3p, miR-29a-3p, miR-126-5p, and miR-375, whereas miR-200a-3p and miR-423-3p were found downregulated. In monitoring patients during follow-up, the authors found that miR-22-3p and miR-29a-3p significantly decreased in postoperative BC patients, and NMIC patients with high miR-22-3p and low miR-200a-3p expression levels had worse recurrence-free survival. Piao et al. [30] identified two miRNAs (miR-6124 and miR-4511) as promising urinary biomarkers for distinguishing nonmalignant hematuria from hematuria associated with BC. The expression ratio between miR-6124 and miR-4511 was found to be significantly higher in patients with BC than in healthy subjects (AUC = 0.865) or subjects with hematuria (AUC = 0.888) or with pyuria (AUC = 0.907). The authors found that both miRNAs could discriminate both NMIBC patients (AUC = 0.855) and MIBC patients (AUC = 0.887) from healthy controls. Looking at the protocols, we identified differences between these two studies [30,37]: protocols for supernatant isolation and the choice of the reference genes. Specifically, Du and colleagues [37] made two steps of centrifugation and the second at a high speed; in contrast, Piao and colleagues [30] made only one centrifugation step at a lower speed. In addition, [37] used as reference genes endogenous miRNAs (miR-532-5p and let-7b-5p), whereas [30] applied the ratio of up-and down-expressed miRNAs. We do not know if these differences could justify different findings, but surely the lack of protocol standardizations should disadvantage homogeneous results.

The remaining six studies [21,26,33,34,40,41], except that of Pardini [33] with a lower diagnostic power (AUC = 0.7), identified miRNA signatures with a diagnostic power ≥ 0.8. Among these, the most promising signature seemed to be that found by [21] with an AUC = 0.92. The uniqueness of this study was to combine surface-enhanced Raman spectroscopy (SERS) with differentially expressed miRNAs (miR-34a-5p, miR-205-3p, and miR-210-3p) to improve the diagnostic power of both alone; this accuracy was higher in both the miRNAs (AUC = 0.84) or SERS data (AUC = 0.84) individually. The accuracy became 0.95 in discriminating luminal vs. basal classifications. Even in these six studies, the lack of standardization procedures recurred once again. As reported in Table 1, the authors used different reference genes: [21,33] chose the same (miR.28-3p and miR-361-3p) genes, but [26] chose exogenous UniSp2; [40] chose RNU6 and RNU48; and [41] chose miR-191, miR-28-3p, and miR-200b; the one used by [34] was not specified. Still, 50% of the studies [26,33,37,40] performed two steps of centrifugation, with the second at a high speed, whereas the remaining studies [21,30,34,41] separated the supernatants performing only one centrifugation step at a moderate speed (Table 1), and the timing and the speeds changed by groups. The centrifugation protocols should have a key role in the supernatant purification. Why did some groups decide to make a second step at ahigh speed and others did not? These evident heterogeneities surely make the results unequal. Therefore, standardization protocols are needed, especially when none of those above studies identified in urine supernatants one or more identical miRNA; the 26 miRNAs were completely different from each other (Supplementary Table S2). Nevertheless, the interesting finding was that miR-205-5p [21,31] was found significantly upregulated and helpful for a BC diagnosis both in whole urine and in the supernatants. Sadly, even if miR423-5p [24,37] and let-7c [16,26] were identified as potential diagnostic biomarkers both in whole urine and in supernatants, the two markers showed a different trend. Specifically, miR-423-5p was found upregulated in whole urine and downregulated in the urine supernatant, and let-7c was found upregulated in urine supernatant and downregulated in whole urine. These discrepancies make us suspect that these results need to be deepened.

### 4.3. miRNA Biomarker Isolated from Urine Sediment

As reported in Table 1, using urine sediment has emerged as one of the two less employed methodological strategies to isolate miRNAs associated with BC (5/25 studies) [20,25,29,36,43]. Among these studies, only [43] took advantage of a discovery cohort (27 BC patients and 58 controls) and validation cohort (61 BC patients and 60 controls). The remaining four studies did not validate the results in a separate independent cohort; overall, the miRNA screening was made on the pellet of 356 BC urine samples and 303 healthy controls urine samples.

The study of Urquidi and colleagues [43] was unique in applying a high-throughput platform for screening miRNAs. They profiled 754 miRNAs on urothelial cell samples of a discovery cohort. A panel of 46 miRNAs significantly associated with BC was subsequently validated in an independent cohort. Multivariable modeling identified a diagnostic panel of 25 miRNAs able to discriminate BC patients with an AUC = 0.98. Among all the 25 studies selected, this impressive signature represents the one with the highest diagnostic power. 

Nevertheless, the results of the study by [25] were noteworthy. In this study, the authors validated a signature of nine miRNA (miR-21, miR-96, miR-125b, miR-126, miR-145, miR-183, miR-205, miR-210, and miR-221), and only six of them (miR-96, miR-125b, miR-126, miR-145, miR-183, and miR-221) showed statistically significant differences between BC patients and the control group (AUC values between 0.605 and 0.772). Combining the diagnostic power of the identified miRNAs with the diagnostic power of the voided urine cytology (VUC), the simultaneous assessment of miR-125b, miR-145, miR-183, miR-221, and VUC reached a diagnostic power of 0.88. 

Between the 25 miRNAs identified by [43] and the 6 miRNAs identified by [25], only miR-96 was shared. 

Eissa’s study [29] should be credited with the originality to assess multiple urinary biomarkers (lncRNA-miRNA-mRNA) to aid in the early detection of bladder cancer. Specifically, they identified a molecular network autophagy-related composed of noncoding RNAs (miR-324-5p, miR-4738-3p, and lncRNA miR-497-HG) and their target genes (RCAN1 mRNA and FOSB mRNA) and assessed their capability to diagnose BC. The authors found that miR-324-5p and miR-4738-3p showed diagnostic powers of 0.883 and 0.815 to discriminate BC patients from the controls, respectively. Nevertheless, FOSB mRNA and RCAN1 mRNA showed the highest accuracy (99% for RCAN1 mRNA or FOSB mRNA). Despite the AUC value of miR-324-5p and miR-4738-3p being lower than the other selected biomarkers, they were higher than cytology (77.6%).

The study by [36] enrolled the lowest cohort compared to the other four studies (46 BC patients and 59 healthy controls). By using bioinformatic tools, the authors selected eight miRNAs (miR-17p, miR-19a-3p, miR-106a-5p, miR-141-3p, miR-145-5p, mir-146a-5p, miR-185-5p, and miR-223-3p) involved in inflammation and the tumor microenvironment. Following their screening in pellet urine of the study cohort, they found that the miR-17-5p, miR-106a-5p, and miR-19a-3p panel was the best model in terms of diagnostic power (AUC = 0.87). This is an interesting result, but as it was determined on a small study cohort, this data should be validated in a separate cohort.

Straddling whole urine, urine sediment, and urine supernatant, there was the study of Hentschel and colleagues [20]. The authors argued the potential of DNA methylation markers as urinary biomarkers for a BC diagnosis. In a previous study, they isolated the nine most discriminative methylation markers, including two miRNAs (FAM19A4, GHSR, MAL, miR-129, miR-935, PHACTR3, PRDM14, SST, and ZIC1) and found that a pellet is the preferred specimen over whole urine and supernatants [49] for diagnostic purposes. The purpose of this study [20] was to validate the results of a previously technical comparative study [49,50]. Using an independent study population (208 participants), they confirmed that the methylation levels of the nine markers in the urine pellet were significantly higher in BC patients than in controls (all, *p* < 0.001). Comparing the AUCs of these nine methylation markers in a urine pellet vs. whole urine, they found a high similarity between the ROC curves of both studies [20,50]. Nevertheless, even if the AUC value of methylated miR-129 and miR-935 were 0.83 and 0.79, respectively, in preclinical validation studies, the authors underlined the appropriate selection of the marker panel GHSR/MAL, which reached an AUC of 0.89 when distinguishing between BC patients and the controls. The authors concluded that both urine pellets and whole urine were suitable for the noninvasive detection of BC.

To conclude, as in the studies mentioned above, the non-reproducibility of the data recurred, and the protocols were different in timing, temperature, and speed. Contrary to the studies cited above, in those that used urine pellets, we found a higher consensus in RNU (4/5 studies) as the reference gene for data normalization (Table 1). The number of miRNAs identified as potential biomarkers for the diagnosis of BC is N = 38 (Supplementary Table S2), very impressive because it is too heterogeneous, and only one was shared in two papers (miRNA-96). Nevertheless, the miRNAs that need attention are miR-221, miR-126, and miR-141, because they have been found related to BC both in sediment and whole urine.

### 4.4. miRNA Biomarker Isolated from Urine Extracellular Vesicles

The use of extracellular vesicles (EVs) in a liquid biopsy is becoming appreciated for its potential to predict different diseases, such as cancer [51]. EVs act as carriers of different biological materials (proteins, lipids, and nucleic acids, including miRNAs) between cells also located in distant regions of the body. Thus, they should result in a representative picture of molecular complexity and heterogeneity of the physiological and pathophysiological processes, such as tumor development. In the systemic revision of the literature, we found four studies [22,23,27,28] published over the past three years assessing the suitability of miRNAs in urinary EVs for a BC diagnosis. As reported in Table 1, the first finding was that the subjects recruited in each study were somewhat smaller than those recruited in the studies focusing on whole urine, supernatants, and pellet.

The study of Baumgart and colleagues [28] analyzed the miRNA expression levels from 32 tissues of NMIBC patients and 50 tissues of MIBC patients, and the best deregulated markers were validated in 20 urine samples of MIBC patients and 17 NMIBC patients. They proposed two EV urinary miRNAs: miR-146b-5p and mi-R-155-5p as a useful diagnostic tool for discriminating MIBC from NMIBC. Both miRNAs were found to be significantly upregulated in urinary EVs from MIBC patients compared with NMIBC patients and associated with the T stage and tumor grade, but the diagnostic power in terms of the AUC was not determined.

El-Shal and colleagues [23] focused their attention on assessing the expression levels of two specific miRNAs: miR-96-5p and miR-183-5p, because they have been found to be dysregulated in breast, ovary, prostate, liver, bladder, and colon cancers [52,53,54]. They screened EVs from urine samples of a study cohort including 51 BC subjects, 21 subjects with benign bladder lesions, and 28 healthy controls. The ROC curve analysis revealed that both miRNAs had greater AUC values (miR-96-5p AUC = 0.85; miR-183-5p AUC = 0.83) than that of cytology (AUC = 0.69) in discriminating BC patients from benign bladder lesions and healthy controls. In addition, combining miR-96-5p with miR-183-5p, the diagnostic performance was raised to 88% (Table 1). In addition, the authors found that the highest values of both miRNAs were correlated with the advancing tumor grade and lymph node invasion, whereas their lowest levels correlated with noninvasive tumors (stages Ta and T1).

Interesting were the results of Güllü and colleagues [27]. For diagnostic purposes, they combined an exosomal miRNA signature with a urinary protein signature and gave promising results. Logistic regression and ROC analyses showed that a panel including EV miRNAs (miR-139, miR-136, miR-19, and miR-210) and EV proteins (BLCA-4, NMP22, APE1/Ref1, CRK, and VIM) were able to discriminate BC patients from healthy subjects with an AUC = 0.903. The same model was able to differentiate low-risk patients from healthy controls with an AUC = 0.976. In low-risk early-stage patients, the panel was more sensitive, suggesting that changes in expression and concentration of the selected molecules could be early events in BC. Nevertheless, as the study was conducted on a limited study cohort (59 BC patients, 34 healthy controls, and 12 patients during follow-up without recurrence), although the results are very interesting, they need to be validated in a larger cohort.

A comprehensive analysis of urinary EV-derived miRNA profiles of BC patients and healthy controls was performed with high throughput technology (NGS) on a discovery cohort including 12 BC subjects (6 NMIBC and 6 MIBC) and 4 healthy controls [22]. The authors found that 51 miRNAs were upregulated and 22 downregulated in BC patients compared to healthy controls; while 40 miRNAs were found upregulated and 21 downregulated when comparing NMIBC patients vs. MIBC patients. Then, the list of obtained differentially expressed miRNAs was intersected with differentially expressed miRNAs in the TCGA database. In this way, only miR-93-5p and miR-516a-5p overexpression were validated in an additional independent cohort of BC patients (53 BC subjects and 51 controls.). The ROC curve analysis showed that both miRNAs had promising AUC values of 0.838 for miR-93-5p and 0.790 for miR-516a-5p. Logistic regression performed by combining both miRNAs showed an improvement in diagnostic accuracy with an AUC value of 0.867, well above that of urine cytology (AUC = 0.630). Moreover, miR-93-5p increased in MIBC compared with NMIBC patients and exhibited promising AUC for distinguishing these two BC stages. The authors performed in vitro functional studies and found that miR-93-5p was involved in cell proliferation and promotes BC cell migration and invasion via inhibiting the target gene BTG2 (B-cell translocation gene 2).

Even in choosing EVs as a biological specimen for BC diagnosis, heterogeneity of the results reoccurred. None of the four studies found the same miRNA, and four papers highlighted the diagnostic power of a total of 10 different miRNAs. Nevertheless, miR-146-5p was found deregulated both in sediment and in extracellular vesicles.

## 5. Conclusions

Bladder cancer is the most common malignancy of the urinary tract. Cystoscopy represents the gold standard for a BC diagnosis, but this procedure is invasive and painful, hence the need to identify new biomarkers with high diagnostic power through noninvasive procedures. Therefore, circulating miRNAs represent an appealing class of molecules for noninvasive diagnostics, but their translation into clinical practice appears to be a future goal. This systematic review highlighted that, although many of the selected studies were of high scientific quality, the results proved to be quite heterogeneous. We did not identify a univocal consensus for a specific miRNA signature but only isolated the signatures, some of which with better diagnostic power compared to the others. We think one of the problems is the lack of standardization in terms of biological samples (whole urine, supernatant, pellet, or EVs) and protocols (centrifugation, reference genes, etc.). Even if urine pellets seem the best choice for miRNAs analyses, as tumor cells can exfoliate and be collected after urine centrifuge, we think that whole urine could represent the most conservative choice. Using whole urine, we can collect all miRNAs regardless of whether they may originate from the pellet or the supernatant. This is important, because we could avoid the analytical pitfall linked to the centrifugation protocols, whether for pellet or supernatant separations. Moreover, the hypothesis that tumor cells in the urine may secrete tumor-derived miRNAs into the urine supernatant cannot be ruled out; thus, the choice of whole urine would favor the analysis of all miRNAs, both those from tumor cells and those secreted by them. An additional suggestion might be to perform, before miRNA screening, an upstream bacterial culture test to be sure that the urine miRNAs are of exclusive human origin. 

To conclude, we believe that there is still a long way to go and that it may be useful in the future, rather than isolating additional miRNA biomarkers for a BC diagnosis, to focus on those that, from this review, represent the most robust in terms of the AUC value by validating them on additional study cohorts.

## Figures and Tables

**Table 1:** Main features of each selected study.

Publication Year (Reference)	Other Biospecimens	Urine Specimen	Centrifuge Protocol	Study Cohort	Method	Reference Gene	miRNA (↑ Increase; ↓ Decrease)	Multiple-miRNA Signature	Diagnostic Power (AUC)
2022 [20]	-	Sediment and whole urine	800× *g* for 10 min at RT	Cohort: BC 108 and CTRL 100	qMSP	ACTB	miR- 129 ↑, miR-935 ↑	no	miR-129 AUC = 0.83 miR-935 AUC = 0.79
2022 [21]	-	Supernatant	3600× *g* for 10 min	Prospective cohort (Romania): BC 15 and CTRL 16 Retrospective cohort (Italy): BC 66 and CTRL 50	NGS and RT-qPCR and SERS	miR-28-3p and miR-361-3p	BC vs. CTRL: miR-34a-5p ↑, miR-205-5p ↑, and miR-210-3p ↑. Luminal vs. basal BC: miR-615-3p ↑ and miR-185-5p ↓	yes	BC vs. CTRL = AUC = 0.92 Luminal vs. Basal = AUC = 0.95
2021 [22]	Tissues and cell lines	Exosome	(1) 3000× *g* for 20 min at 4 °C (2) 17,000× *g* for 30 min and filtered 0.22 μm (3) 110,000× *g* for 70 min at 4 °C	Discovery Cohort: BC 12 (6 NMIBC and 6 MIBC) and CTRL 4. Validation Cohort: BC 53 and CTRL 51	NGS and RT-qPCR	exogenous cel-miR-39 and U6 snRNA	miR-93-5p ↑ and miR-516a-5p ↑	yes	AUC= 0.87
2021 [23]	Serum	Exosome	(1) 3000× *g* for 30 min (2) 13,000× *g* for 5 min at 4 °C (3) Kit for Exosomal purification	Cohort: BC 51, Benign urinary Bladder Lesions (BL) 21 and CTRL 28	RT-qPCR	SNORD68	miR-96-5p ↑ and miR-183-5p ↑	yes	miR-96-5p Sens = 80.4 Spec = 91.8 AUC = 0.85 miR-183-5p Sens = 78.4 Spec = 81.6 AUC = 0.83; miR-96-5p + miR-183-5p Sens = 88.2 Spec = 87.8 AUC = 0.88; miR-96-5p + cytology Sens = 82.4 Spec = 91.8 AUC = 0.87; miR-183-5p + cytology Sens = 80.4 Spec = 91.8 AUC = 0.85
2021 [24]	-	Whole urine	miRNeasy Serum/Plasma kit	Discovery cohort: BC 10 and CTRL 10.Validation cohort: BC 80 and CTRL 100.	NGS and RT-qPCR	not reported	let-7b-5p ↑, miR-149-5p ↑, miR-146a-5p ↑ and miR-423-5p ↑	-	not reported
2020 [25]	-	Sediment	(1) 1500× *g* for 10 min at 4 °C (2) 870× *g* for 5 min at 4 °C	Cohort: BC 104 and CTRL 46	RT-qPCR	RNU44 and RNU48	miR-96 ↑, miR-125b ↓, miR-126 ↑, miR-145 ↓, miR-183 ↑, and miR-221 ↓	yes	miR-125b + miR-145 + miR-183 + miR-221 + VUC Sens = 84,6%, Spec = 95.7% AUC = 0.88
2020 [26]	Tissue	Supernatant	(1) 4500 rpm for 30 min at 4 °C (2) 8900 rpm for 5 min at 4 °C	Cohort: BC 57 and CTRL 20	RT-qPCR	exogenous UniSp2	let-7c ↑	no	AUC = 0.80
2020 [27]	-	Exosome	(1) 1000 rpm for 10 min at 4 °C (2) 2500 rpm for 10 min at 4 °C	Cohort: BC 59 and CTRL 34 and Follow-up patients without recurrence 12	RT-qPCR	RNU48 and RNU6	miR-19b1-5p ↑, miR-136-3p ↑, miR-139-5p ↑, miR-210-3p ↑	yes	Sens = 80.0%, Spec = 88.2%, AUC = 0.903
2019 [28]	Tissue	Exosome	(1) 2000× *g* for 20 min at 4 °C (2) 15,000× *g* for 30 min at 4 ° C	Discovery cohort: Tissue NMIBC 10 and MIBC 14; Validation Cohort: Tissue NMIBC 22 and MIBC 36. Urine samples MIBC 20 and NMIBC 17	Microarray and RT-qPCR	RNU48	miR-146b-5p ↑ and miR-155-5p ↑	-	not reported
2019 [29]	Plasma and tissue	Sediment	4000 rpm for 10 to 20 min at R.T.	Cohort: BC 98 and BL 48 and CTRL 50	RT-qPCR	RNU6	miR-324-5p ↑ and miR-4738-3p ↑	no	BC vs. all not BC: miR-324-5p Sens = 87.8, Spec = 86.7, AUC = 0.883 miR-4738-3p Sens = 84.7, Spec = 80.6, AUC = 0.827 BC vs. benign cases: miR-324-5p Sens = 76.5, Spec = 89.6, AUC = 0.801 miR-4738-3p Sens = 76.5, Spec = 93.8, AUC = 0.815 NMIBC vs. MIBC miR-324-5p Sens = 49.4, Spec = 46.7, AUC = 0.490 miR-4738-3p Sens = 48.2, Spec = 46.7, AUC = 0.490 Low-grade BC miR-324-5p Sens = 58.9, Spec = 40.0, AUC = 0.531 miR-4738-3p Sens = 58.9, Spec = 44, AUC = 0.551
2019 [30]	-	Supernatant	2500 rpm for 15 min at 4 °C	Discovery Cohort: BC 35 BC and CTRL 20 Training Cohort: BC 174 and CTRL 114Validation Cohort: BC 117 and CTRL 97	Microarray, RT-qPCR	Ratio of up- and down expressed miRNAs	miR-6124 ↑ and miR-4511 ↓	yes	BC vs. CTRL Sens = 91.5, Spec = 74.2, AUC = 0.865 NMIBC vs. CTRL Sens = 88.9, Spec =77.3, AUC = 0.855 MIBC vs. CTRL Sens = 94.4, Spec = 74.2, AUC = 0.887
2019 [31]	-	Whole urine	-	Cohort: BC 45 and CTRL 23 and Bbenign prostatic hyperplasia (BPH) 22	RT-qPCR	5S rRNA	miR-21-5p ↑, miR-141-3p ↑, and miR-205-5p ↑	no	miR-21-5p Sens = 84.0, Spec =59.0, AUC = 0.76 miR-141-3p Sens = 71.0, Spec = 71.0, AUC = 0.74 miR-205-5p Sens = 82.0, Spec = 62.0, AUC = 0.73
2018 [16]	-	Whole urine	-	Discovery cohort: BC 8 [low-grade BC (LGNMIBC) and 4 high-grade BC (HGNMIBC)] and CTRL 8 Validation cohort: BC 115 (56 LGNMIBC, 34 HGNMIBC, 25 MIBC) and CTRL 87	Microarray and RT-qPCR	RNU48 and miR-103	let-7c ↓, miR-148a ↓, miR-204a ↓, miR-135a ↑, miR-135b ↑, miR-345 ↑	yes	BC vc CRTL AUC = 0.88LGNMIBC vs. CTRL AUC = 0.88 HGNMIBC vs. CTRL AUC = 0.93 MIBC vs. CTRL AUC = 0.91
2018 [32]	-	Whole urine	-	Cohort: BC 66, CTRL 53	RT-qPCR	5S rRNA	miR-10b ↑, and miR-34b ↑, miR-103 ↑, miR-141 ↓	yes	Sens = 75.0, Spec = 63.5, AUC = 0.72
2018 [33]	-	Supernatant	(1) 3000× *g* for 10 min (2) 12,000× *g* for 10 min at 4 °C	Discovery cohort: BC 66 and CTRL 48. Validation cohort: BC 46 and CTRL 16.	NGS and RT-qPCR	miR-28-3p and miR-361-3p	Model 1: age, smoking status and miR-30a-5p ↓, let-7c-5p ↓, miR-486-5p ↑	yes	AUC = 0.7
2018 [34]	-	Supernatant	2000× *g* for 15 min at 4 °C	Cohort: BC 205 and CTRL 99 and non-metastatic clear-cell renal cell carcinoma 30	Microarray and RT-qPCR	not specified	miR-93-5p ↑, miR-31-5p ↑	yes	Sens = 74.0, Spec =75.0, AUC = 0.81
2017 [35]	Tissue and blood	Whole urine	-	Cohort: BC 76 and CTRL 66	RT-qPCR	U6	miR-186 ↓	-	not reported
2017 [36]	-	Sediment	2000× *g* for 10 min at 4 °C	Cohort: BC 46 and CTRL “0” 28 and CTRL “1” (bladder inflammation) 31	RT-qPCR	RNU6	miR-141-3p ↓, miR-19a-3p ↑ miR-17-5p ↑, miR-106a-5p ↑	yes	HG vs. LG BC miR-17-5p AUC = 0.57; HR vs. LR BC miR-17-5p AUC = 0.61, miR-19a-3p AUC = 0.60; NMI-LG vs. NMI-HG miR-17-5p AUC = 0.63; BC vs. CTRL miR-17-5p + miR-106a-5p + miR-19a-3p AUC = 0.87
2017 [37]	-	Supernatant	(1) 1500× *g* for 10 min at 4 °C (2) 13,800× *g* for 15 min at 4 °C	Discovery cohort: BC 6 and CTRL 6 Training cohort: BC 150 and CTRL 150 Validation Cohort: BC 120, CTRL 120	NGS and RT-qPCR	miR-532-5p and let-7b-5p	miR-7-5p ↑, miR-22-3p ↑, miR-29a-3p ↑, miR-126-5p ↑, miR-200a-3p ↓, miR-375 ↑, and miR-423-5p ↓	yes	Logistic regression analysis Sens = 85.0 %, Spec = 86.7 %, AUC: 0.92)
2017 [38]	-	Whole urine	-	Cohort: BC 134 and CTRL 268 (urinary tract infection, UTI)	RT-qPCR	RNA U6	miR-126 ↑	-	not reported
2016 [39]	Cell lines and tissues	Whole urine	-	Cohort: BC 28, CTRL 10 with UTI, CTRL 19	Microarray and RT-qPCR	miR-21-5p	miR146a-5p ↑	no	Sens = 100, Spec = 53.5, AUC = 0.77
2016 [40]	Tissue	Supernatant	(1) 3000× *g* for 10 min (2) 16,000× g for 10 min at 4 °C	Discovery cohort: BC 30 and CTRL 30 Validation Cohort: BC 162 and Cystitis 76 and CTRL 86	RT-qPCR-Direct	U6 and RNU48	miR-155 ↑	no	Sens = 80.2, Spec = 84.6 AUC = 0.80
2016 [41]	-	Supernatant	4000 rpm for 10 min at 10 °C	Discovery cohort: BC 46 and CTRL 13 Validation Cohort: BC 27 and CTRL 23	Microarray and RT-qPCR	miR-191, miR-28-3p and miR-200b	miR-125b ↓, miR-30b ↓, miR-204 ↓, miR-99a ↓, and miR-532-3p ↓	no	BC vs. CTRL miR-125b Sens = 59.26, Spec = 95.65, AUC = 0.801 miR- 99a Sens = 74.07, Spec = 82.61, AUC = 0.738 miR-204 Sens = 53.85 Spec = 100 AUC = 0.771 miR-30b Sens = 66.67 Spec = 82.61 AUC = 0.760 miR-532-3p Sens = 59.26 Spec = 86.96 AUC = 0.818
2016 [42]	-	Whole urine	-	Discovery cohort: BC 30 and Previous BC affected but now without recurrence 30 and CTRL 21 Validation cohort: BC 25 and CTRL 25	RT-qPCR	urine osmolarity	miR-16 ↑, miR-200c ↑, miR-205 ↑, miR-21 ↑, miR-221 ↑, miR-34a ↑	yes	BC Recurrence vs. BC without recurrence Sens = 80.0, Spec = 48.0, AUC = 0.74; CTRL vs. T1 stage: AUC = 0.92
2016 [43]	-	Sediment	600× g for 5 min at 4 °C	Discovery cohort: BC 27 and CTRL 58 Validation cohort: BC 61 and CTRL 60	Microarray and RT-qPCR	RNU6	↑: miR-652, miR-199a-3p, miR-140-5p, miR-93, miR-142-5p, miR-224, miR-96, miR-766, miR-223, miR-99b, miR-140-3p, let-7b, miR-141, miR-191, miR-146b-5p, miR-491-5p, miR-339-3p, miR-200c, miR-106b*, miR-143, miR-429, miR-222, miR-200a ↓: miR-1305, miR-30a	yes	Sens = 87.0, Spec = 100.0, AUC = 0.982

Abbreviations: BC (bladder cancer), NMIBC (non-muscle invasive bladder cancer), MIBC (muscle invasive bladder cancer), BL (bladder lesion), CTRL (control), qMSP (quantitative methylation-specific PCR), NGS (next generation sequencing), RT-qPCR (reverse transcription quantitative real-time PCR), SERS (surface-enhanced Raman spectroscopy), UTI (urinary tract infection), Sens (sensibility), Spec (specificity), and AUC (area under the curve).

**Table 2:** Tabular presentation for the QUADAS-2 results.

Study	Risk of Bias							Applicability Concerns		
	Patient Selection		Index Test		Reference Standard	Flow and Timing		Patient Selection	Index Test	Reference Standard
	**Was a Consecutive or Random Sample of Patients Enrolled?**	**Did the Study Avoid Inappropriate Exclusions?**	**Were the Index Test Results Interpreted without Knowledge of the Results of the Reference Standard?**	**If a Threshold was Used, was it Pre-specified?**	**Is the Reference Standard Likely to Correctly Classify the Target Condition?**	**Was There an Appropriate Interval between Index Test and Reference Standard?**	**Did All Patients Receive the Same Reference Standard?**	**Are There Concerns That the Included Patients and Setting Do Not Match the Review Question?**	**Are There Concerns That the Index Test, Its Conduct, or Interpretation Differ from the Review Question?**	**Are There Concerns That the Target Condition as Defined By the Reference Standard Does Not Match the Question?**
**Study 1**	**+**	**+**	**?**	**+**	**+**	**?**	**+**	**+**	**+**	**+**
**Study 2**	**+**	**+**	**?**	**?**	**+**	**?**	**+**	**+**	**+**	**+**
**Study 5**	**+**	**+**	**?**	**-**	**?**	**?**	**+**	**+**	**+**	**+**
**Study 7**	**+**	**+**	**?**	**+**	**+**	**?**	**+**	**+**	**+**	**+**
**Study 8**	**+**	**?**	**?**	**-**	**?**	**?**	**?**	**+**	**-**	**+**
**Study 11**	**+**	**+**	**?**	**+**	**+**	**?**	**+**	**+**	**+**	**+**
**Study 14**	**+**	**+**	**?**	**?**	**+**	**+**	**+**	**+**	**+**	**+**
**Study 16**	**+**	**+**	**?**	**-**	**+**	**+**	**+**	**+**	**+**	**+**
**Study 19**	**?**	**+**	**?**	**-**	**+**	**?**	**+**	**+**	**-**	**-**
**Study 20**	**+**	**+**	**?**	**+**	**?**	**?**	**-**	**+**	**+**	**+**
**Study 22**	**+**	**+**	**?**	**-**	**+**	**?**	**+**	**+**	**+**	**+**
**Study 23**	**+**	**+**	**?**	**+**	**?**	**?**	**-**	**-**	**+**	**+**
**Study 24**	**+**	**+**	**?**	**+**	**+**	**+**	**+**	**+**	**+**	**+**
**Study 25**	**+**	**+**	**?**	**-**	**+**	**+**	**+**	**+**	**+**	**+**
**Study 26**	**+**	**+**	**?**	**?**	**+**	**+**	**+**	**+**	**+**	**+**
**Study 28**	**+**	**+**	**?**	**-**	**+**	**+**	**+**	**+**	**+**	**+**
**Study 29**	**+**	**+**	**?**	**-**	**?**	**?**	**?**	**+**	**-**	**-**
**Study 30**	**+**	**+**	**?**	**-**	**+**	**?**	**+**	**+**	**+**	**+**
**Study 33**	**+**	**+**	**?**	**+**	**+**	**+**	**+**	**+**	**+**	**+**
**Study 34**	**+**	**+**	**?**	**+**	**?**	**?**	**?**	**+**	**-**	**-**
**Study 36**	**+**	**+**	**?**	**-**	**?**	**?**	**?**	**+**	**+**	**+**
**Study 37**	**+**	**?**	**?**	**?**	**+**	**+**	**+**	**+**	**+**	**+**
**Study 38**	**+**	**+**	**?**	**-**	**+**	**?**	**+**	**+**	**+**	**+**
**Study 39**	**+**	**+**	**?**	**+**	**+**	**?**	**+**	**+**	**+**	**+**
**Study 41**	**+**	**+**	**?**	**+**	**+**	**+**	**+**	**+**	**+**	**+**

**+** Low risk; **-** High risk; **?** Unclear risk.

**Table 3:** miRNAs, linked to the 25 eligible studies, were divided for their finding.

Found in One Study (66 miRNAs)	Found in Two Studies (15 miRNAs)	Found in Three Studies (1 miRNA)
let-7b, miR-103, miR-135b, miR-136-3p, miR-141, miR-146b-5p, miR-17-5p, miR-19a-3p, miR-204, miR-205, miR-221, miR-29a-3p, miR-30b, miR-93, miR-185-5p, miR-34b, miR-106a-5p, miR-106b*, miR-10b, miR-126-5p, miR-129, miR-1305, miR-135a, miR-139-5p, miR-141, miR-142-5p, miR-143, miR-145, miR-146b-5p, miR-148a, miR-16, miR-17-5p, miR-186, miR-191, miR-199a-3p, miR-19a-3p, miR-19b1-5p, miR-204a, miR-221, miR-222, miR-223, miR-22-3p, miR-224, miR-31-5p, miR-324-5p, miR-339-3p, miR-345, miR-375, miR-423-5p, miR-429, miR-4511, miR-4738-3p, miR-486-5p, miR-491-5p, miR-516a-5p, miR-532-3p, miR-6124, miR-615-3p, miR-652, miR-7-5p, miR-766, miR-935, miR-99a, miR-99b, miR-140-3p, miR-140-5p	Let7-c/let-7c-5p, miR-125b, miR-126, miR-141-3p, miR146a-5p, miR-155/miR-155-5p, miR-200a/miR-200a-3p, miR-205-5p, miR-210-3p, miR-30a/miR-30a-5p, miR-34a/miR-34a-5p, miR-93-5p, miR-200c, miR-21/miR-21-5p, miR-183/miR-183-5p	miR-96/miR-96-5p

## Data Availability

Not applicable.

## Supplementary Materials

The following supporting information can be downloaded at: https://www.mdpi.com/article/10.3390/biomedicines10112766/s1. Listing S1: Key Terms Used in Literature Search; Table S1: PRISMA Checklist; Table S2: miRNAs found in urine specimens.
